# Antenatal exposure to second hand smoke of non-smoking mothers and growth rate of their infants

**DOI:** 10.1371/journal.pone.0218577

**Published:** 2019-06-20

**Authors:** Frida Soesanti, Cuno S. P. M. Uiterwaal, Diederick E. Grobbee, Aryono Hendarto, Geertje W. Dalmeijer, Nikmah Salamia Idris

**Affiliations:** 1 Department of Child Health, Faculty of Medicine, Universitas Indonesia/Cipto Mangunkusumo General Hospital, Jakarta, Indonesia; 2 Julius Center for Health Sciences and Primary Care, Julius Global Health, University Medical Center, Utrecht, the Netherlands; Hopital Robert Debre, FRANCE

## Abstract

**Objectives:**

There is limited evidence on the effect of exposure to second hand smoke (SHS) in non-smoking pregnant mothers and infant health. We assessed the effects of maternal antenatal exposure to SHS on infant growth rate, and secondarily, on birth weight, birth length and head circumference at birth.

**Methods:**

In this prospective cohort, 305 mother-infant pairs were studied. Mothers filled out questionnaires about exposure to SHS in pregnancy at the 3^rd^ trimester of pregnancy. Infant anthropometry was performed at birth, day 7, and months 1, 2, 4, and 6, postnatally. Linear mixed modeling and linear regression were used to calculate growth rates over the first 6 months. The association between SHS-exposure with growth rate and birth sizes was assessed using multivariate linear regression adjusted for confounders, with SHS as both number of cigarettes and as groups (no exposure, SHS < 23 cigarettes, SHS ≥ 23 cigarettes).

**Results:**

Seventy-three mothers were not exposed and 232 were exposed. SHS exposure (per cigarette) was not related to gain in weight, length, head circumference, and weight for length. However, infants born to mothers exposed to ≥ 23 cigarettes/d had lower head circumference gain (-0.32 mm/m, 95% CI -0.60, -0.03) than those born to non-exposed mothers. SHS exposure (per cigarette) was not related to birth weight, length, and head circumference, but exposure to ≥ 23 cigarettes was related to lower head circumference at birth (-11.09 mm, -20.03, -2.16).

**Conclusions:**

Heavy antenatal exposure to SHS in non-smoking mothers results in reduced neonatal head circumference at birth and head circumference gain over the first 6 months of life. Our findings show no clear relations between exposure to SHS during pregnancy and other markers of neonatal growth and birth size.

## Introduction

Tobacco smoke is one of the most ubiquitous environmental health hazards not only for adults but also for children.[1] Infants from mothers who smoked 15 or more cigarettes per day during pregnancy had lower weight and smaller head circumference at birth and continued to be smaller until the age of 2 years compared to children of non smoking mothers.[2] Prenatal exposure to second hand smoke (SHS) involves exposure to the same range of tobacco smoke toxins experienced by active smokers, but at lower levels.[3,4] Therefore, it is likely that exposure to SHS also causes some or all of complications caused by active smoking but with lower levels of relative risk.[3,4] Prenatal exposure to SHS is reported to be associated with higher risk of still birth (23%), congenital malformation (13%),[4] and lower birth weight,[3,5–7] also reported to be associated with shorter body length and decreased head circumference (HC) at birth, [3,8–11] and higher adiposity in childhood period.[12–14]

Health consequences of SHS-exposure during pregnancy were mostly studied in developed countries where SHS-exposure is generally lower and environmental conditions (pollution and ventilation) are better than in developing countries. For this reason, the effects of SHS-exposure on infant health may be more pronounced in low to middle-income countries (i.e., Indonesia), making it a major public health concern. Worldwide, it has been reported that 35% of female non-smokers are exposed to SHS.[1] This high prevalence of SHS exposure is largely the result of high smoking prevalence among men. [1] In Indonesia, pregnant women are frequently exposed to SHS as a result of overcrowding and poor ventilation, making this a unique setting to study the effects of SHS exposure on infant growth. There is substantial evidence on the effect of maternal smoking during pregnancy on child growth,[14–16] and obesity,[14] and only one study stated that exposure to SHS during infancy leads to weight and height growth reduction in the first four months of life.[8] However, not many studies were done on the effect of exposure to SHS specifically during pregnancy on the growth of their offspring in infancy.

We aimed to assess the effects of maternal antenatal exposure to SHS on infant growth rate. Secondarily, we aimed to estimate the association between maternal exposure to second hand smoke during pregnancy with birth weight (BW), birth length and head circumference.

## Material and methods

This is a cohort study based on data obtained in the ongoing Breastfeeding Attitude and Volume Optimization (BRAVO) trial (NCT01566812) performed in Jakarta, Indonesia.[17] The BRAVO study was ethically approved by the Institutional Review Board of the Faculty of Medicine University of Indonesia/Cipto Mangunkusumo General Hospital, Jakarta, Indonesia (reference number: 913/UN2.F1/ETIK/X/2012).[17]

Pregnant women were recruited during their antenatal care visit at the third trimester in three centers i.e. one hospital specialized in maternal and child care (Budi Kemuliaan Hospital, Jakarta) and two primary care centers in Senen and Jatinegara district, Jakarta, Indonesia. Pregnant women were included in the BRAVO study if they were residing in the vicinity of the participating hospitals or primary care centers, telephone communication with mother was possible, had uncomplicated pregnancy (no morbidity requiring hospitalization or intensive care), no detected major fetal congenital disease, and were not known to have HIV.[17] Recruitment was performed by midwives or primary care workers in charge of routine pregnancy care from July 2012 until early 2017. Eligible pregnant women were first informed about BRAVO study and they were given one week to decide and were asked to inform their midwives or primary care physicians about their decision. Pregnant women who decided to participate in the study were asked to sign BRAVO informed consent form.[17] Subjects were included in the present analysis if they had complete data on SHS exposure and at least two anthropometric measurements of their offspring, between birth and 6 months of age.

Maternal characteristics including maternal age, parity, history of abortion, work status, family income, level of education, body mass index (BMI), weight gain during pregnancy (expressed as Δ BMI: BMI at labor-BMI prior to pregnancy), alcohol or illicit drug use in pregnancy were obtained from self-reported questionnaires filled out by pregnant women at recruitment.

Gestational age at delivery (including preterm birth), Apgar score, maternal hypertension and diabetes (pre-existing or gestational), specifics of neonatal morbidity (special nursery care requirements, including sepsis, respiratory distress, and hyperbilirubinemia) were obtained from medical records.

### SHS exposure of mothers during pregnancy

Pregnant women who did not smoke but were exposed to household members who smoked on a daily basis at home in the presence of the pregnant women were considered as exposed to SHS during pregnancy. Exposure to SHS during pregnancy was determined by self-reported questionnaires filled out by the pregnant women at recruitment. Smoking habits during pregnancy of pregnant women, their husband and other house hold members were recorded. Data regarding the average number of cigarettes smoked per day throughout the duration of pregnancy for each smoker, pregnancy period (trimester) when the exposure happened, and whether the household member smoked in the presence of the pregnant women were obtained. No data was available for postnatal smoking behavior of household members.

### Growth rate and birth size measurement

Birth weight and length were measured at birth using the standardized protocol by the midwife who helped in the delivery process. Infant weight and length and HC were measured at birth, 7 days, 1 month, 2 months, 4 months, and 6 months of age, reported in days post-delivery.

As growth is linear during the first six postnatal months, infants with at least two measurements available within that period were included in analyses, unless only measurements at birth and day 7 were available.[18,19] Subsequently, linear mixed modeling was performed with extraction of estimated random slopes per child for weight, length, and head circumference. Linear regression was performed to calculate the predicted values per child, giving the estimated length gain rate (reflecting lean mass accumulation), weight gain rate and HC increment per child.[18,19] Weight gain rate was expressed as weight gain per day, while height gain rate and HC increment were expressed per month. Weight gain rate adjusted for length gain rate (WLG) was assessed (to reflect excess weight gain) for each child by deriving Z-score internal in our study population and calculating the standardized residuals from the linear regression model with weight gain as the dependent variable and length gain as the independent variable.[18,19]

### Confounders/Effect modification

Level of education, household income,[20] maternal age, maternal BMI (ΔBMI), parity, and breastfeeding were a priori considered as possible confounders.[4,10,14,21,22] Mothers were asked to fill in a breastfeeding diary during the follow-up period, specifying during what part of the follow-up period they performed exclusive breastfeeding. Breastfeeding was considered as an effect modifier.[23] Other explored effect modifiers were total number of cigarettes per day consumed by household members and BW category (low or normal).

### Statistical analysis

Baseline characteristics were tabulated by exposure to SHS during pregnancy (exposed and non-exposed). Continuous variables were expressed as mean and standard deviation or median and interquartile range if distributions were skewed. Group differences were estimated and tested by independent groups t-test, chi-square test, or Fisher’s exact test where appropriate and p values were provided. We excluded the actively smoking mothers from the final analysis.

The association of total cigarettes consumed by household members with growth rate and birth sizes was analyzed using multivariable linear regression adjusted for confounders. Also, in order to evaluate effects of extreme SHS exposure we categorized the exposure to SHS into three groups based on the distribution of exposure to cigarettes. The most extreme exposure category was defined as the median of the cigarettes exposure (12 cigarettes) + upper quartile range (10 cigarettes), thus we had the non-exposed to SHS (NE) group, the SHS exposed group with < 23 cigarettes/day (SHS <23 group), and the SHS exposed group with ≥ 23 cigarettes/day (SHS ≥ 23 group). Multivariate linear regression adjusted for confounders was used to assess the association between SHS-exposure and growth rate (weight, length, HC, and WLG) and also between SHS-exposure and birth sizes (weight, length, HC). To that end, dummy variables were created for SHS < 23 and SHS ≥ 23 cigarettes and simultaneously entered into the models as independent variables.

The possibility of interaction between BW and SHS on weight gain rate and WLG rate were tested by adding a product term of BW Z-Score and SHS group (BW*SHS) to the models. We tested the interaction between BW and total number of cigarettes exposure (expressed by internal Z-score) on gain rate and WLG rate to the models. We also explored the possibility of modification by breastfeeding with SHS and total number of cigarettes exposure on growth rate. Statistical significance was assumed if 95% confidence intervals did not include the estimation null values, corresponding to two-sided p values <0.05. Statistical analyses were conducted using IBM SPSS version 24 for Mac.

## Results

There were 735 children who had finished the 6 months period of follow-up. Three hundred and five children were included in this study, where the remaining were excluded due to incomplete SHS exposure measurement or incomplete growth measurement. Around 76% of mothers were exposed to SHS during pregnancy. Non-SHS exposed mothers were slightly older and had higher socio-economic status (SES) compared to the SHS exposed. Six mothers were smoking throughout the pregnancy period. The main source of exposure was fathers (86.3%) who consumed 6 cigarettes/day on average, while 35.5% of subjects were exposed to more than one source of SHS. No difference was found for the breastfeeding rate up to 6 months between both groups (Table 1).

**Table 1:** Baseline subjects characteristics.

Variable	No SHS exposure (n = 73)	SHS exposure (n = 232)	p-value
**INFANT**			
**Male sex, n (%)**	40 (54.8)	119 (52.0)	0.66
**Gestational age (weeks)**	38.9 (1.2)	38.9 (1.4)	2.1 0.85
**Breastfed until 4 months, n (%)**	59 (81.0)	180 (77.0)	0.63
**Breastfed until 6 months, n (%)**	51 (70.0)	147 (63.0)	0.90
**Formula at birth, n (%)**	8 (12.7)	25 (12.7)	0.97
**FAMILY**			
**Maternal age (years)**	29.2 (5.4)	28.0 (5.6)	0.16
Parity*	2 (2)	2 (2)	0.88
**History of abortion, n (%)**	8 (12.3)	28 (13.4)	0.12
**BMI mother before pregnancy (kg/m^2^)**	22.4 (4.0)	22.5 (4.7)	0.90
**BMI increment by pregnancy (kg/m^2^)**	3.6 (2.2)	3.9 (2.6)	0.43
**Family income (USD/month), n (%)**			
• **> 223**	17 (23.9)	32 (14.0)	0.002
**Educational level mother, n (%)**			
• **University**	11 (15.3)	12 (4.8)	0.02
• **Senior high school**	45 (63.9)	157 (68.6)	
• **Elementary-junior high school**	16 (20.8)	61 (26.6)	
**SMOKING STATUS DURING PREGNANCY**			
**Mother smoking, n (%)**	0	6 (2.6)	
• Cigarettes/day*	0	11 (3)	
**Father smoking, n (%)**	0	200 (86.3)	
• Cigarettes/day*	0	6 (9)	
**Number of household members smoking, n (%)**			
• **1 member**	0	152 (65.5)	
• **2 members**	0	54 (23.3)	
• **3 members**	0	16 (6.9)	
• **4 members**	0	10 (4.3)	
Total cigarettes per day*	0	12 (10)	

Numbers are expressed in mean (SD) otherwise indicated

*: median (interquartile range).

Table 2 shows the association between total number of cigarettes exposure with postnatal growth rate. During the postnatal period, number of cigarettes consumed by household was inversely associated with the HC increment per month. Gain rates of weight, length, and WLG were increased with higher number of antenatal cigarettes exposure, but none of these relations were statistically significant.

**Table 2:** Associations between exposure to household SHS in non-smoking mothers during pregnancy and growth rate markers.

		Linear regression coefficients (95% confidence intervals)			
Continuous	Model	Weight gain (g/day)	Length gain (mm/month)	Head circumference gain (mm/month)	WLG (Z-score)
Number of cigarettes exposure	Crude	0.02 (-0.03,0.07)	0.004 (-0.004, 0.012)	-0.009 (-0.018, 0.000)	0.004 (-0.004, 0.020)
	Adjusted^a^	0.01 (-0.04, 0.06)	0.005 (-0.003, 0.012)	-0.008 (-0.017, 0.001)	0.003 (-0.012, 0.018)
**Categorical**					
Non-exposed		Reference	Reference	Reference	Reference
SHS <23 (n = 195)	Crude	-0.50 (-1.58, 0.57)	-0.11 (-0.28, 0.06)	-0.11 (-0.31, 0.08)	-0.17 (-0.49, 0.17)
	Adjusted^a^	-0.60 (-1.69, 0.49)	-0.10 (-0.27, 0.06)	-0.13 (-0.32, 0.07)	-0.19 (-0.52, 0.14)
SHS ≥23 (n = 37)	Crude	0.14 (-1.39, 1.67)	0.18 (-0.05, 0.43)	-0.34 (-0.62, -0.07)	0.07(-0.39, 0.53)
	Adjusted^a^	0.03 (-1.54, 1.60)	0.18 (-0.06, 0.43)	-0.32 (-0.60, -0.03)	0.03 (-0.45, 0.50)

^a^Adjusted for delta BMI, SES, parity, breastfeeding at 6 month.

In the categorical analysis (Table 2), infants in SHS ≥ 23 group had statistically significantly lower HC increment per month than the NE group (-0.32 mm/month, p = 0.03). This difference was not found in the SHS < 23 group. Infants in the SHS ≥ 23 group had higher but non-statistically significant gain rates of weight, length and WLG compared to the NE group. Infants born to mothers in the SHS < 23 group had lower growth rates of weight, length, head circumference and WLG, but none of these were statistically significant.

Table 3 shows that the number of cigarettes consumed by household members was inversely but not statistically significantly associated with birth sizes i.e., BW, birth length, and HC. Table 3 shows in the categorical analysis, that infants in the SHS ≥ 23 group had significantly smaller HC than those born to NE mothers (-11.09 mm, p = 0.02). There was no significant difference in BW and birth length between the three groups, but the BW in the SHS ≥ 23 group was 55.9 grams lower than the SHS <23 group and 97.6 grams lower than the NE group.

**Table 3:** Associations between exposure to household SHS in non-smoking mothers during pregnancy and birth sizes.

		Linear regression coefficients (95% confidence intervals)		
Continuous	Model	Birth weight (g)	Birth length (mm)	Head circumference at day 5 (mm)
Number of cigarettes exposure	Crude	-2.0 (-7.1, 3.0)	-0.1 (-0.4, 0.1)	-0.2 (-0.5, 0.1)
	Adjusted^a^	-2.3 (-7.6, 2.9)	-0.1 (-0.4, 0.1)	-0.3 (-0.5, 0.0)
**Categorical**				
Non-exposed		Reference	Reference	Reference
SHS < 23	Crude	-38.69 (-149.99, 72.60)	-0.35 (-6.46, 5.76)	-1.01 (-7.33, 5.32)
(n = 195)	Adjusted^a^	-40.72 (-158.96, 77.52)	-0.30 (-6.34, 5.74)	-1.35 (-7.65, 4.94)
SHS ≥ 23	Crude	-108.08 (-269.39, 53.22)	-2.91 (-11.40, 5.63)	-10.06 (-18.95, -1.17)^b^
(n = 37)	Adjusted^a^	-97.59 (-269.08.97, 73.90)	-4.82 (-13.24, 3.60)	-11.09 (-20.03, -2.16)^b^

^a^ adjusted for delta BMI, SES, parity

^b^ statistically significant

There was no interaction found between SHS exposure and Z-score of BW on weight gain rate (interaction term coefficient (β) of 0.31 and 0.44, p> 0.05, respectively) or WLG (β of 0.06 and 0.11, p>0.05, respectively). There was also no interaction found between number of cigarettes consumed by household and BW on weight gain rate and WLG rate (interaction term coefficient of -0.07 and -0.23, p> 0.05, respectively). Furthermore, there was no modification effect of breastfeeding status on weight gain rate, height gain rate, HC increment and WLG rate (all product term p-values were > 0.05).

### Mothers who were smoking during pregnancy

All mothers who smoked during pregnancy were also exposed to SHS during pregnancy. Infants from these mothers were born smaller than infants of NE and SHS exposed group, with a mean BW of 2900 grams (-244 grams of the NE group and -175 grams of the SHS exposed group). These infants also had lower birth length (46.25 cm) and head circumference (33 cm). Their growth rate was not different from the non-exposed and SHS exposed group.

## Discussion

This study provides evidence regarding the effect of maternal antenatal exposure to SHS on birth sizes and infant growth rate. We observed no material relations between SHS exposure in pregnancy with BW and length nor on weight gain and length gain over the first 6 months of life. Infants born to non smoking mothers who had higher exposure to SHS (≥ 23 cigarettes per day) had significantly lower HC at birth and lower HC increment than the NE group. To our knowledge, ours is the first study to evaluate the effect on infant growth of exposure to SHS in mothers who do not smoke during pregnancy. Previous studies on the topic focused on effects of active maternal smoking during pregnancy on perinatal outcome or on postnatal growth of the infant.

We consider the prospective cohort design a relative strength, where some other studies used self-reporting questionnaires or telephone interviews to measure smoke exposure retrospectively.[12,14] We took self-reports of pregnancy smoking data during the 3rd pregnancy trimester which minimizes SHS misclassification. Our primary outcome measures, markers of infant body growth, were taken using standard protocols by research personnel that was unaware of mothers’ questionnaire data, specifically SHS exposure, nor were participants aware of the research hypothesis. We believe that our design makes information bias an unlikely explanation for our findings. Although we cannot exclude the possibility of residual confounding, we did measure all a-priori known confounding factors and adjusted for them. A particular strength of our study is its specific Indonesian setting with smoke exposure originating almost exclusively from mothers’ surroundings. In Indonesia, around 70% of men smoke, versus only 3% of women,[1] and indeed in our study, 76% of pregnant non-smoking mothers were exposed to SHS during pregnancy and the main source of exposure was their husband. We do believe that this lends credibility for SHS, being the culprit for any adverse health effects in the children.

Like other studies, we did use self-reporting to obtain the information on antenatal exposure to SHS.[2,16,24] Other researchers have measured the cotinine level in cord blood or other specimens,[9,25] but we were not able to obtain such biomaterials. Technical errors in anthropometrics measurement may have occurred but if any we assume that this was independent of exposure. Our cohort size is substantial for the Indonesian context, but it may be too small to statistically detect all effects. We did not collect data on postnatal exposure to SHS which might have a role in postnatal growth, while we assume that postnatal SHS exposure is likely a continuation of prenatal SHS exposure. Our study did not find any modification of effect by breastfeeding status on any of growth markers. Finally, we could not adjust for SHS exposure from office or public space due to lack of data on these exposures.

Head circumference is known as an indicator of abnormal brain condition or neurodevelopmental delay in intelligence and cognitive function.[24,26] A study stated that weight and HC were significantly smaller in neonates whose mothers smoked ≥ 15 cigarettes/day, but the difference disappeared by 3 years of life while they continued to lag behind in length growth until the age of 6 years.[2] Another study showed that at 6 months of age, infants who were born to mothers who smoked during pregnancy had lower weight and HC compared to those of non-smoking mothers.[24,27] Exposure to SHS in non-smoking pregnant women was reported to be associated with 0.24 cm reduction in HC of the newborn compared to NE group.[25] Active smoking of mothers antenatally was reported to be related to a 0.13 mm/week reduction of fetal HC increment compare to non-smoking mothers and this diminished HC appeared to persist throughout early childhood.[28] This result may be in line with the findings of our present study, showing that HC of the infant grows slower postnatally when non-smoking mothers were heavily exposed to SHS (≥ 23 cigarettes per day). We believe that our findings are consistent with the proposition that heavy exposure to SHS during pregnancy may well have a similar order of magnitude of effect as with active maternal smoking in pregnancy.

Our main finding that SHS exposure in pregnancy slows HC growth in early infancy, and possibly in utero, may reflect hampered brain development. However, mechanisms underlying cigarette smoke toxin, especially nicotine effects on human brain development are incompletely understood.[24,26,28] Animal studies showed that nicotine directly influences fetal brain development, even in concentrations that do not cause growth retardation.[24,26,28] Prenatal exposure to nicotine causes altered cell proliferation and differentiation which results in cell damage, cell loss and synaptic dysfunction. In humans, higher rates of behavioral problems were found in mothers who smoked during pregnancy.[24,26,28] The consequences of subnormal HC are well-known in (very) low BW children,[29] this indicator of brain volume is negatively associated with cognitive function and neuropsychological abilities at early school age.[26,29]

There were no clear associations between antenatal maternal exposure to SHS and postnatal infant weight gain rate, length gain rate, and WLG rate. A reduction on BW and length was noticed in mothers who were exposed to higher number of cigarettes but this was not statistically significant. This is not fully in line with previous studies, that showed a consistent reduction of 31–79 grams in BW[3,5–7] and exposure to SHS during pregnancy was also reported to reduce birth length.[25]

In summary, the results of this study support the view that antenatal SHS exposure to non-smoking mother results in reduced HC at birth and diminished catch-up, postnatally. As decreased HC has been associated with delay in neurodevelopment and reduce cognitive function, long term follow-up is needed to evaluate the effect of head circumference growth on neurodevelopmental and cognitive function. Although antenatal maternal exposure to SHS does not clearly affect other post natal growth parameter, there was an indication that exposure to SHS in mothers during pregnancy has some effect detrimental on infant birth sizes and infant growth. Repeating this analysis in a larger study may be worthwhile. Real action should be implemented to reduce the harmful effect of SHS exposure to mothers during pregnancy to their offspring by increasing the awareness in the society.

## Supporting information

S1 Dataset Data for antenatal exposure to SHS in non-smoking mother and the anthropometric measurements of the infant. (SAV)
